# False positive urine drug test for tricyclic antidepressants attributed to quetiapine treatment

**DOI:** 10.1192/j.eurpsy.2025.1517

**Published:** 2025-08-26

**Authors:** E. Routsi, M. Kousounadis, L. Lymperopoulos, N. Kokras

**Affiliations:** 1 First Department of Psychiatry, National and Kadopistrian University of Athens, Eginition Hospital, Athens, Greece

## Abstract

**Introduction:**

Quetiapine is a serotonin-dopamine antagonist widely used for the treatment of bipolar disorder and schizophrenia. Patients with bipolar disorder and schizophrenia are also at high risk of drug abuse. Illicit substances are often traced in such patients during manic and psychotic episodes. Urine drug tests are commonly used to detect illicit substance use during hospital admissions.

**Objectives:**

We report a case of an adult male patient treated with 600mg o.d. quetiapine who falsely tested positive for tricyclic antidepressants in a routine urine drug test

**Methods:**

A male patient with a prior history of alcohol, cannabis and cocaine abuse acutely developed psychotic-like symptoms (persecutory delusions, psychomotor retardation, social withdrawal) and attempted to commit suicide. He was admitted to our psychiatric hospital, and he denied illicit drug use during the last 5 months.

**Results:**

The patient was treated with quetiapine monotherapy, progressively titrated up to 600mg o.d. As a routine procedure and because of his personal history of drug abuse, he has been subjected to a urine drug test, which revealed positive results for tricyclic antidepressants. Due to uncertainty whether he abused tricyclic antidepressants prior to this admission, a second test was ordered after two weeks of quetiapine monotherapy and close inpatient monitoring, which was also positive for tricyclic antidepressants.

**Conclusions:**

Quetiapine has a three-ringed chemical structure which shares similarities with tricyclic antidepressants. in vitro tests proved cross-reactivity of quetiapine and tricyclic antidepressants with some commercially available immunoassays. However it is not clear if the cross-reactivity is due to quetiapine or its active metabolites. In any case, the interpretation of a urine test positive for tricyclic antidepressants should take into account the possibility of such cross-reactivity with quetiapine, especially in cases of suspected drug overdose when the urine test is used to deduct the possible offending drug. Moreover, this cross-reactivity might be eploited in cases of suspected non-adherence to quetiapine treatment.

**Disclosure of Interest:**

None Declared

